# Effect of Preinfection Health Status on COVID‐19 Severity and Cognitive Function

**DOI:** 10.1002/brb3.70793

**Published:** 2025-10-07

**Authors:** Kang Yuan, Wenbiao Xian, Lishan Lin, Fengjuan Su, Feiwen Huang, Wenli Sheng, Wanling Wu

**Affiliations:** ^1^ Department of Neurology, The First Affiliated Hospital, Sun Yat‐sen University; Guangdong Provincial Key Laboratory of Diagnosis and Treatment of Major Neurological Diseases; National Key Clinical Department and Key Discipline of Neurology Guangzhou Guangdong PR China; ^2^ Nursing Department Hui Ya Hospital of The First Affiliated Hospital, Sun Yat‐Sen University Huizhou Guangdong PR China

## Abstract

**Background:**

This observational cohort study investigates how infection health factors influence COVID‐19 severity and cognitive outcomes. We collected preinfection data from hospitalized COVID‐19 patients, including demographic information and baseline health conditions prior to diagnosis, and examined their associations with hospitalization duration and cognitive function assessed after infection.

**Methods:**

Data were obtained from Hui Ya Hospital, The First Affiliated Hospital, Sun Yat‐sen University, China. The study included confirmed COVID‐19 patients requiring hospitalization. Among the 147 collected cases, two were excluded due to missing data, leaving a final sample of 145 patients. The Montreal Cognitive Assessment (MoCA), which evaluates global cognitive function with a total score of 0–30, was used to assess cognitive function, while hospitalization duration and routine clinical examinations were analyzed as indicators of disease severity. Additionally, the SF‐12v2 score reflecting Health‐Related Quality of Life was used to evaluate patients' overall health status. Statistical analyses were conducted to identify preinfection factors associated with COVID‐19 outcomes.

**Results:**

Preinfection baseline health status was significantly correlated with both hospitalization duration (*p* < 0.0001, 95% CI [−0.47, −0.16]) and MoCA scores (*p* = 0.0001, 95% CI [0.15, 0.46]). Patients with better preinfection health conditions experienced shorter hospital stays and demonstrated better cognitive function postinfection.

**Conclusion:**

Our findings indicate that preinfection baseline health conditions play a crucial role in determining both the severity of COVID‐19 and postinfection cognitive function. Specifically, impairments were more pronounced in the visuospatial, naming, attention, calculation, language, and memory domains. Additionally, our results suggest a potential link between COVID‐19 outcomes and patients’ preexisting underlying diseases.

## Graphical abstract

Baseline health status, as measured by the SF‐12v2, was a strong predictor of shorter hospital stay and better postinfection cognitive outcomes. The presence of comorbidities was associated with increased disease severity and significant impairments in attention and language domains, highlighting the importance of proactive health management to reduce associated risks.

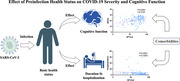

## 1 Introduction

COVID‐19 is a severe form of acute respiratory distress syndrome and lung inflammation caused by infection with the novel coronavirus SARS‐CoV‐2 (Zhu et al. 2020). According to data from the World Health Organization, until February 2025, there have been 777 million confirmed cases of COVID‐19 with 7.08 million reported deaths worldwide. In China alone, 99 million people have been infected, resulting in 122,398 deaths (World Health Organization 2025). Given the substantial proportion of the global population affected by COVID‐19, understanding its transmission, impact, and outcomes remains a critical public health priority.

SARS‐CoV‐2 can spread primarily through aerosol transmission (Sharma et al. 2021), enabling asymptomatic, presymptomatic, and symptomatic individuals to transmit the virus via respiratory droplets. Additionally, transmission can occur through contact with contaminated surfaces or inanimate objects (Cai et al. 2020). The most common symptoms of COVID‐19 include fever, cough, and dyspnea (Wiersinga et al. 2020). Additionally, individuals who have recovered from COVID‐19 remain susceptible to reinfection (Flacco et al. 2022).

The increasing number of COVID‐19 cases has placed a significant burden on healthcare systems, straining hospital resources and limiting the availability of medical care. In some countries, even hotels have been repurposed as isolation facilities to accommodate patients (Bruni et al. 2020; Fenton et al. 2020). More severe symptoms and worse clinical outcomes necessitate greater medical resources, underscoring the importance of identifying factors that influence disease severity and prognosis. Preexisting health conditions have been shown to affect COVID‐19 outcomes, with evidence indicating that individuals with comorbidities such as hypertension, diabetes, obesity, asthma, and chronic kidney disease are at higher risk of developing severe disease (Gao et al. 2021). Additionally, studies suggest that COVID‐19 infection can impair cognitive function (Ceban et al. 2022).

Given these findings, we know that COVID‐19 can cause lasting damage to human health and cognitive function, and the threat of this infection is still present today, so it is important to understand the conditions that affect the prognosis of this disease and explain their relevance. Thus, it is essential to explore how patients' preinfection health status influences COVID‐19 outcomes and cognitive function. By examining the correlation between baseline health conditions, hospitalization duration, and postinfection cognitive function scores, this study aims to determine the extent to which preinfection health status affects disease severity and neurological outcomes and explore the underlying mechanism in it.

## 2 Methods

### 2.1 Study Design and Participants

This observational cohort study analyzed data from 147 patients admitted to Hui Ya Hospital, The First Affiliated Hospital, Sun Yat‐sen University. All enrolled patients were diagnosed with COVID‐19 and required hospitalization.

### 2.2 Data Collection and Assessment Scales

To conduct the cohort study, patient data were collected using standardized assessment scales. Data collection occurred between February 1, 2023, and February 5, 2023. The assessments included preinfection factors such as baseline health status and comorbidities, as well as postinfection outcomes, including disease severity and cognitive function. These variables were quantified during hospitalization using standardized scoring systems to facilitate analysis.

### 2.3 Baseline Health Status Assessment

Baseline health status was assessed using a modified version of the SF‐12v2 (the Medical Outcomes Study Short‐Form Health Survey) (Hayes et al. 2017; Lam et al. 2013). This scale evaluates eight health‐related domains: physical functioning, role‐physical, bodily pain, general health, vitality, social functioning, role‐emotional, and mental health, collectively reflecting Health‐Related Quality of Life (Shah et al. 2018). The individual item scores range from 0 to 100, where 0 denotes the poorest health and 100 the best. The scores of both PCS (physical component summary) and MCS (mental component summary) subscales are calculated from the survey's 12 questions, and the overall baseline health score is calculated as the average of these items.

### 2.4 Disease Severity and Cognitive Function Assessment

COVID‐19 severity was assessed by hospitalization duration, with longer stays serving as an indicator of greater disease severity. Postinfection cognitive function was evaluated using the Montreal Cognitive Assessment (MoCA) (Hoops et al. 2009). Each patient received a corresponding cognitive function score based on these assessments.

### 2.5 Data Management

Patient questionnaire data were digitized and managed using Microsoft Excel (Microsoft Corp., Redmond, WA, USA), ensuring systematic storage and retrieval of assessment scores.

### 2.6 Statistical Analysis

Correlation analysis was performed to examine the relationship between baseline health scores, hospitalization duration, and MoCA scores, as well as the association between biochemical indicators and these clinical outcomes. Then, propensity score matching (PSM) analysis was used to further explore the association between baseline health scores, hospitalization duration, and MoCA scores with patients grouped by the median health score. Patients were also stratified into groups based on the presence of underlying diseases. Independent *t*‐tests were conducted to compare health scores and MoCA scores between groups, alongside biochemical marker analysis. Odds ratios and 95% confidence intervals (CIs) were calculated.

All statistical tests were two‐tailed, with *p*‐values < 0.05 considered statistically significant. Statistical analyses were conducted using GraphPad Prism 9 (GraphPad Software Inc., San Diego, CA) and R software (version 4.4.3).

## 3 Results

**FIGURE 1:**
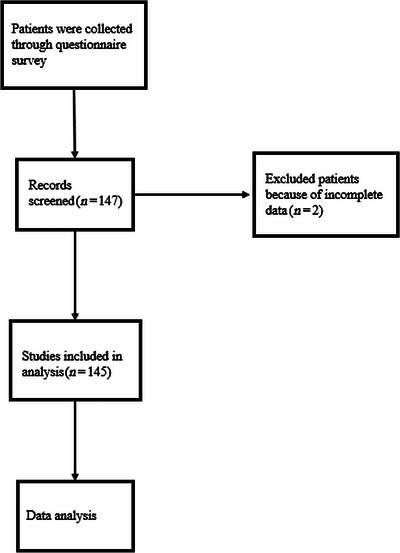
Flow diagram of the study selection process

During the study period, a total of 148 patients were assessed, of whom 145 met the inclusion criteria and were included in the final analysis (Figure 1). Clinical data collected for these patients encompassed demographic information (age, gender, years of education), physical activity levels (exercise and energy expenditure), baseline health status, hospitalization duration, preexisting conditions, and accompanying COVID‐19 symptoms. The demographic characteristics of the study population are summarized in Table 1. The average age of the included patients was 49 years, with 61 males and 84 females, yielding a male‐to‐female ratio of approximately 10:7. The distribution of preexisting conditions is also presented. Among these, hypertension was the most prevalent comorbidity, affecting 34 patients, while asthma and chronic obstructive pulmonary disease were the least common, each reported in only one patient. Other frequently observed conditions included diabetes (20 cases), kidney disease (21 cases), and musculoskeletal disorders (11 cases), while the remaining conditions affected fewer than 10 patients.

**TABLE 1:** Demographic characteristics and comorbidities

Characteristics	Mean or patients (*n* = 145)
Age Male/female Year of education Length of hospitalization Exercise and energy expenditure Basic health condition	49.74 (18.33) 61 (42)/84 (58) 10.60 (3.35) 6.53 (8.65) 68.53 (69.26) 68.13 (18.93)
Stroke	5 (3.40)
Coronary heart disease	8 (5.50)
Diabetes	20 (13.80)
Hypertension	34 (23.40)
Asthma	1 (0.70)
Chronic obstructive pulmonary disease	1 (0.70)
Kidney disease	21 (14.50)
Liver disease	3 (2.10)
Gastrointestinal chronic disease	6 (4.10)
Muscle and bone disease	11 (7.60)
Depression	2 (1.40)
Anxiety	4 (2.80)

*Note*: Data are *n* (%) or mean (SD). Percentages are calculated by category after exclusion of missing data for the variable.

**FIGURE 2:**
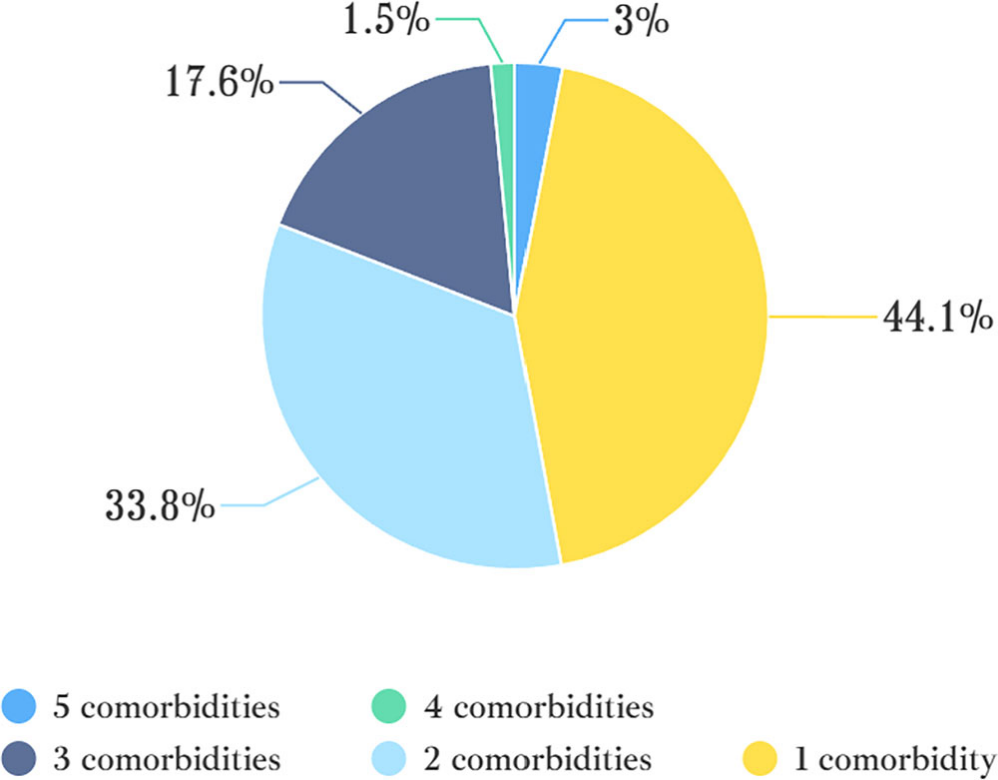
Proportion of patients in different comorbidity numbers

Regarding COVID‐19 symptoms (Table 2), fever was the most frequently reported symptom (115 patients), whereas myocarditis was the least common, affecting only one patient. Symptoms observed in more than 50 patients included fever (115), chills (80), dry cough (85), productive cough (87), nasal congestion (80), runny nose (69), fatigue (94), myalgia or joint pain (83), sore or dry throat (79), and headache (72). These findings highlight the most common symptoms experienced by COVID‐19 patients. In addition, the proportion of patients with multiple coexisting diseases is illustrated in a pie chart (Figure 2).

**TABLE 2:** Accompanying symptoms of patients included in the present study

Accompanying symptoms	Patients (*n* = 145)
Fever	115 (79.30)
Chills	80 (55.20)
Dry cough	84 (57.90)
Coughing up phlegm	87 (60.00)
Coughing up blood	12 (8.30)
Stuffy nose	80 (55.20)
Runny nose	69 (47.60)
Shortness of breath/difficulty breathing	41 (28.30)
Pneumonia	17 (11.70)
Tiredness	94 (64.80)
Sore muscles or joints	83 (57.20)
Dry or sore throat	79 (54.50)
Headache	72 (49.70)
Nausea/vomiting	29 (20.00)
Diarrhoea	21 (14.50)
Loss of taste	45 (31)
Loss of smell	23 (15.90)
Palpitations, rapid heartbeat	27 (18.60)
Myocarditis	1 (0.70)

*Note*: Data is *n* (%). Percentages are calculated by category after exclusion of missing data for the variable.

To further investigate the impact of various pre‐COVID‐19 factors on postinfection outcomes, we performed correlation analyses between multiple preinfection scale scores and postinfection indicators, including length of hospital stay and MoCA scores. These analyses revealed a significant correlation between patients' baseline health status and both hospitalization duration and MoCA scores (*p* < 0.05). Additionally, linear regression analyses were conducted for these two pairs of variables to identify potential trends.

**FIGURE 3:**
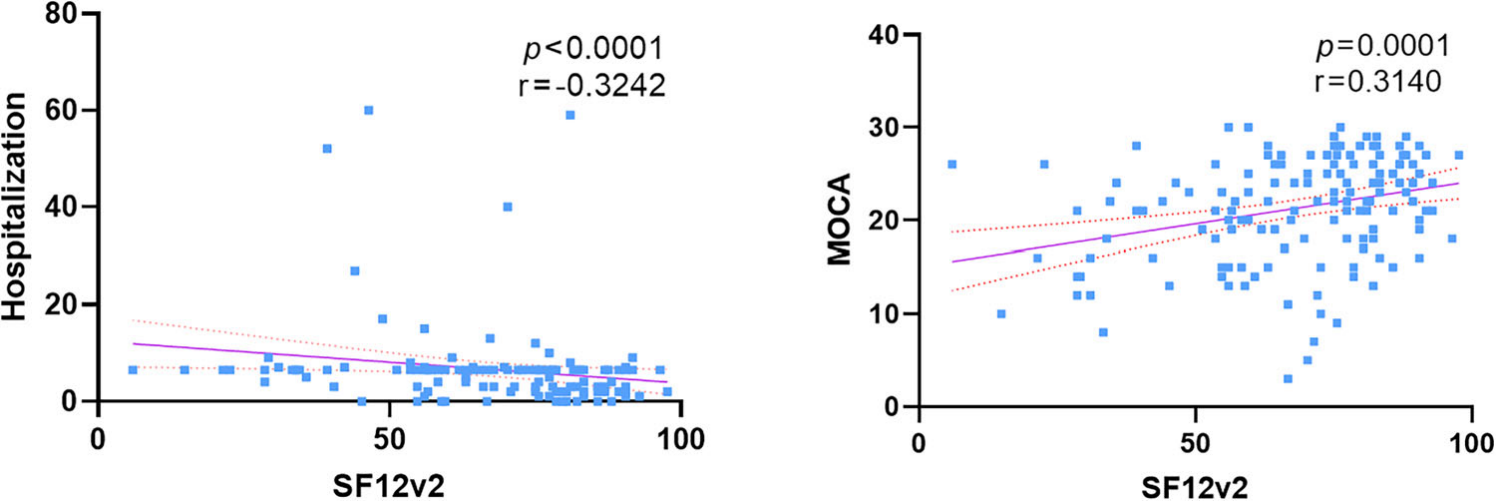
Correlation analysis between health, MOCA, and time of hospitalization

We analyzed the relationship between baseline health status and hospitalization duration (Figure 3). For patients with missing hospitalization data, mean‐value imputation was applied. Consequently, a total of 145 patients were included in this correlation analysis, and some patients were hospitalized for a long time because of their severe condition, which resulted in individual excessive length of hospitalization. A strong negative correlation was observed between baseline health status and hospitalization length (*p* < 0.0001), indicating that poorer preinfection health status was associated with more severe disease progression post‐COVID‐19.

Next, we examined the correlation between baseline health status and MoCA scores. Patients with missing baseline health or MoCA scores were excluded, resulting in 145 cases for this analysis. The correlation analysis demonstrated a significant association between baseline health status and cognitive function postinfection (*p* < 0.0001).

Based on these findings, we infer that better preinfection health status is associated with improved cognitive function and shorter hospitalization duration following COVID‐19 infection.

**FIGURE 4:**
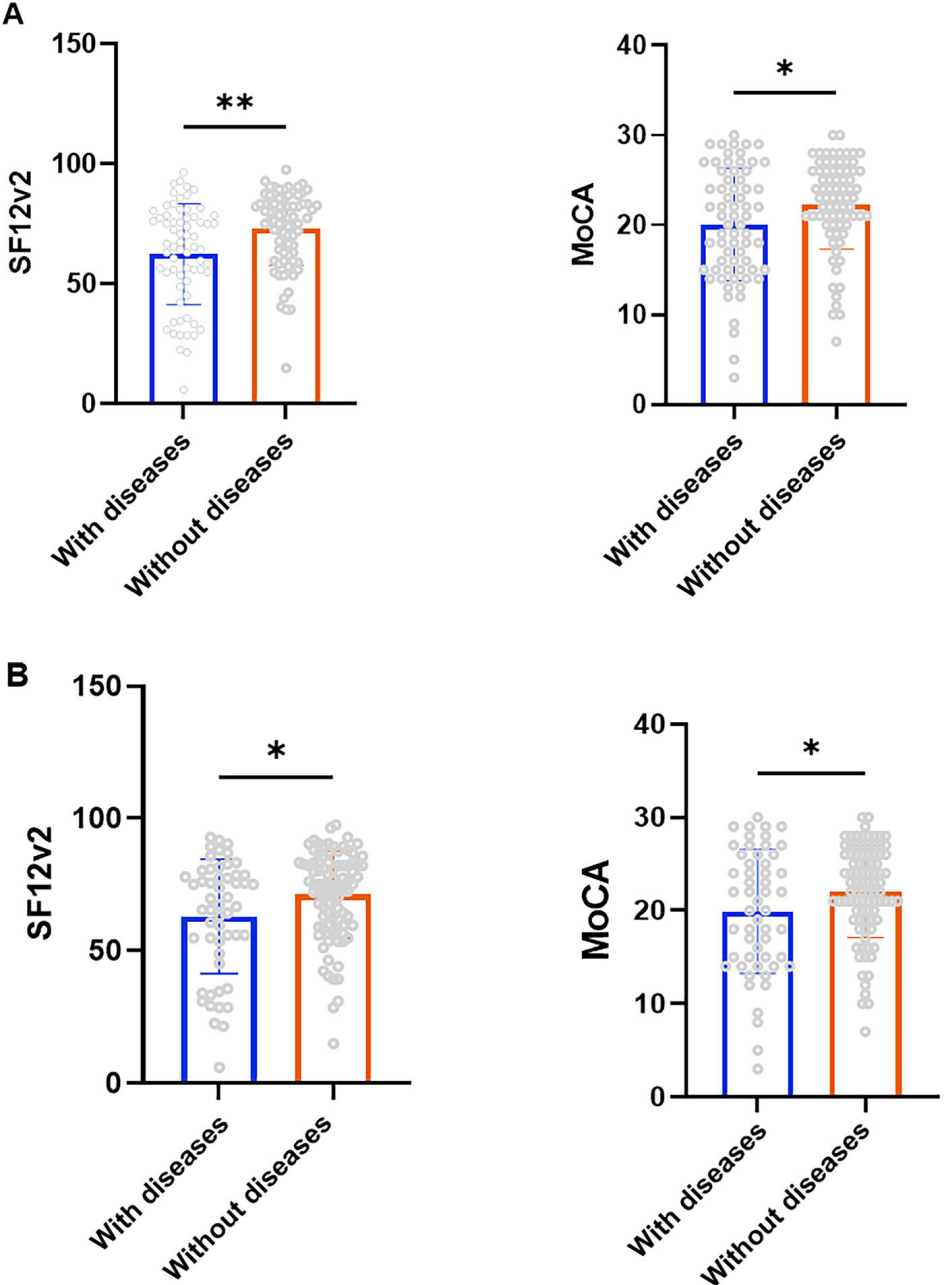
(A) *t*‐Test in classification of all underlying diseases (all diseases existed before infection); (B) t‐test in classification of cognitive‐related underlying diseases (include stroke, coronary heart disease, diabetes, and hypertension). *Note*: ns means *p*‐value> 0.05 and the data are not statistically significant. *p ≤ 0.05, **p ≤ 0.01, ***p ≤ 0.001.

To further explore whether underlying health conditions influenced COVID‐19 severity and cognitive function, we conducted a subgroup analysis (Figure 4). At the same time, we reclassified stroke, coronary heart disease, diabetes, and hypertension as key comorbidities influencing cognitive function and performed subgroup analyses accordingly (Figure 4). The results indicated that patients with preexisting conditions had significantly lower baseline health status and cognitive function compared to those without underlying diseases.

In order to examine the advanced associations among patients’ baseline health status, post‐illness duration of hospitalization, and cognitive function, PSM was performed with SF‐12v2 dichotomized at its median. After matching, the high‐health‐score group (SF‐12v2 ≥ median) demonstrated a significantly higher MoCA score than the low‐health‐score group, whereas no significant difference in length of hospitalization was observed in Table 3.

**TABLE 3:** PSM analysis on SF‐12v2 with MoCA and time of hospitalization

	Variables	Estimate	Standard error	*t*‐value	*p*‐value
MoCA	(Intercept)	18.758	2.126	8.824	< 0.001^***^
	Treat	4.085	1.448	2.821	0.005^**^
	SF‐12v2	0.006	0.038	0.161	0.873
Hospitalization	(Intercept)	10.371	3.429	3.024	0.003^**^
	Treat	−1.698	2.336	−0.727	0.468
	SF‐12v2	−0.044	0.062	−0.707	0.481

*p ≤ 0.05, **p ≤ 0.01, ***p ≤ 0.001, and the data in bold are statistically significant.

We also analyzed the effects of baseline health status on different cognitive domains (Table 4). The results revealed that not all MoCA domains were significantly associated with health scores. The domains exhibiting significant correlations included visuospatial, naming, attention, calculation, language, and memory.

**TABLE 4:** Correlation analysis of health and different domains of MoCA

	Correlation	
Domain	*r*‐value	*p*‐value
Visuospatial	0.1674	0.0449^*^
Naming	0.2238	0.007^*^
Attention	0.3518	<0.0001^*^
Calculation	0.2164	0.0092^*^
Language	0.2287	0.0058^*^
Abstraction	0.1358	0.1046
Memory	0.2014	0.0155^*^
Orientation	0.1456	0.0816

*Note*: * means that *p*‐value ≤0.05, and the data in bold are statistically significant.

**FIGURE 5:**
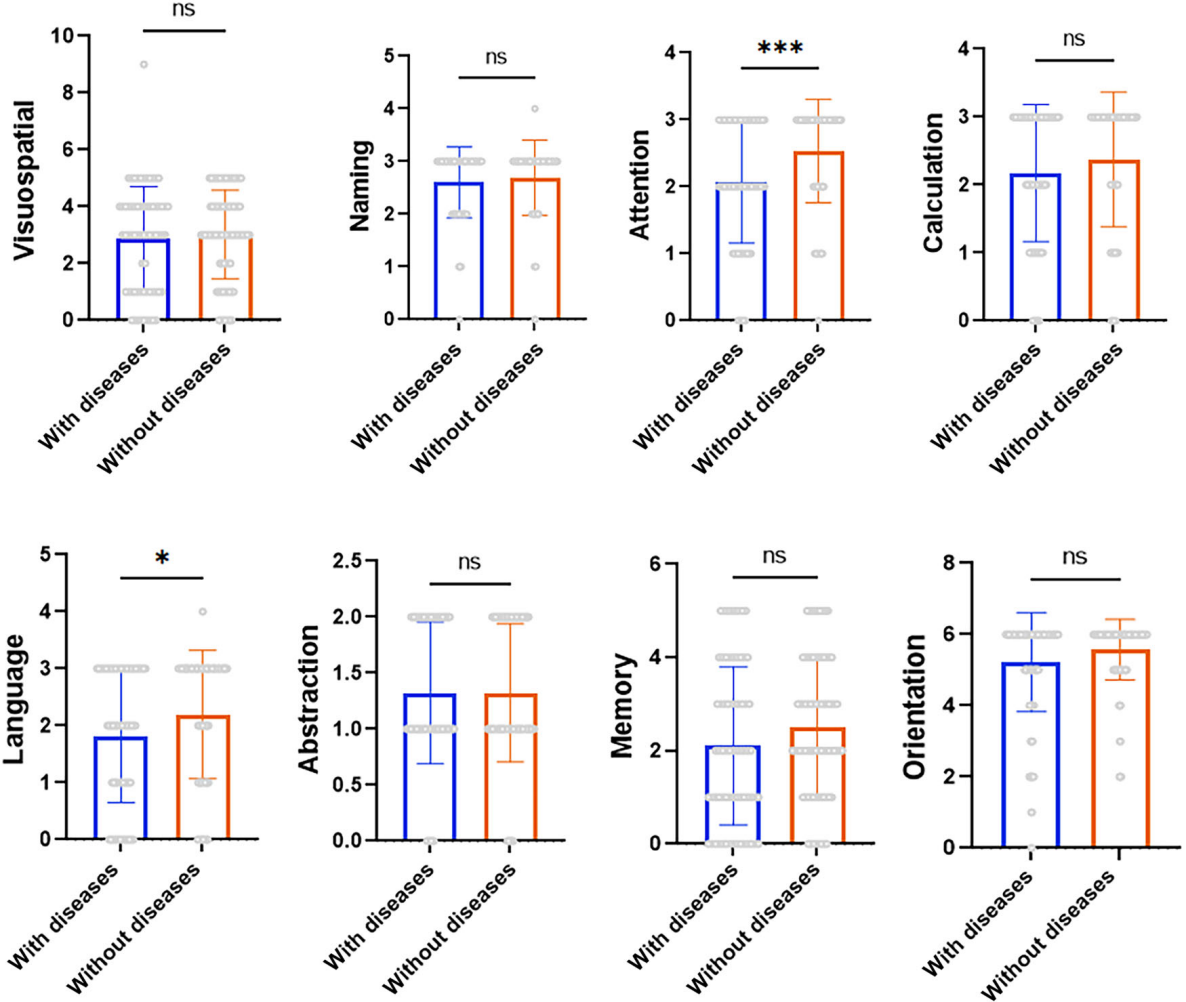
*t*‐Test results of MoCA domains after grouping for the presence or absence of disease. *Note*: ns means *p*‐value> 0.05, and the data are not statistically significant. *p ≤ 0.05, **p ≤ 0.01, ***p ≤ 0.001.

Additionally, we examined the correlation between specific preexisting conditions and individual MoCA domains (Figure 5). The findings suggest that underlying diseases predominantly affect the attention and language domains.

A further analysis was conducted on the impact of underlying diseases on biochemical indicators (Table 5). Group analysis revealed significant differences in Scr (serum creatinine) and systolic blood pressure between patients with and without an underlying condition.

**TABLE 5:** t‐Test analysis of laboratory test results between patients with and without disease

Laboratory characters	p‐value
Hb (g/L)	0.8441
PLT (×10^9^/L)	0.2989
WBC (×10^9^/L)	0.1803
Lymphocyte (g/L)	0.3049
Albumin (umol/L)	0.2507
Bilirubin (U/L)	0.6091
AST (U/L)	0.2003
ALT (U/L)	0.3853
Scr (umol/L)	0.0147^*^
Systolic blood pressure (mmHg)	0.0057^*^
Diastolic blood pressure (mmHg)	0.0921
Blood glucose (mmol/L)	0.2674
LDL (mmol/L)	0.0890
HDL(mmol/L)	0.4921
Triglycerides (mmol/L)	0.2029
Total cholesterol (mmol/L)	0.5876

*Note*: * means that *p*‐value ≤0.05, and the data in bold are statistically significant.

Furthermore, some biochemical indicators exhibited strong correlations with MoCA scores, baseline health status, or hospitalization length. The significant correlations observed were with white blood cell (WBC) count, albumin, aspartate aminotransferase (AST), diastolic blood pressure, low‐density lipoprotein (LDL), triglycerides, and cholesterol levels (Table 6).

**TABLE 6:** Correlation analysis results between laboratory indicators and MOCA, health, and length of stay

	MoCA						SF‐12v2						Hospitalization					
	Total (144)		With disease (68)		Without disease (77)		Total		With disease		Without disease		Total		With disease		Without disease	
	*r*‐value	*p*‐value	*r*‐value	*p*‐value	*r*‐value	*p*‐value	*r*‐value	*p*‐value	*r*‐value	*p*‐value	*r*‐value	*p*‐value	*r*‐value	*p*‐value	*r*‐value	*p*‐value	*r*‐value	*p*‐value
Hb (g/L)	0.1512	0.0695	0.1706	0.1643	0.1617	0.1601	0.02462	0.7696	0.1138	0.359	−0.08457	0.4646	−0.05958	0.4766	−0.1671	0.1733	0.04406	0.7036
PLT (×10^9^/L)	−0.03277	0.6955	0.06427	0.6026	−0.08461	0.4644	0.04684	0.5772	0.08066	0.5164	0.08277	0.4742	0.04713	0.5735	−0.02559	0.8359	0.06879	0.5522
WBC (×10^9^/L)	0.04508	0.5903	0.1661	0.1758	−0.1183	0.3056	0.02893	0.7307	0.1096	0.3774	−0.1001	0.3865	−0.167	0.0447^*^	−0.04007	0.7456	−0.2723	0.0166^*^
Lymphocyte (×10^9^/L)	0.08152	0.3297	0.07119	0.564	0.07909	0.4941	0.1449	0.0832	0.1844	0.1352	0.0891	0.4409	−0.09619	0.2498	−0.1418	0.2489	−0.02263	0.8451
Albumin (g/L)	0.1415	0.0897	0.2663	0.0282^*^	−0.005895	0.9594	0.08441	0.3144	0.3156	0.0093^*^	−0.1153	0.3181	−0.04062	0.6276	−0.2477	0.0417^*^	0.1805	0.1162
Bilirubin (umol/L)	0.01007	0.9043	0.08567	0.4873	−0.1113	0.3351	0.05113	0.5428	0.1329	0.2837	−0.06853	0.5537	−0.0837	0.3169	−0.07547	0.5407	−0.09644	0.4041
AST (U/L)	−0.1152	0.1676	−0.04608	0.7091	−0.1513	0.1889	−0.01697	0.84	0.1212	0.3286	−0.1016	0.3793	0.09067	0.2781	0.2886	0.017^*^	−0.114	0.3236
ALT (U/L)	−0.01256	0.8808	0.1318	0.284	−0.1657	0.1499	0.03439	0.6824	0.1351	0.2757	−0.04332	0.7083	0.04349	0.6035	0.1427	0.2457	−0.09305	0.4209
Scr (umol/L)	0.009702	0.9078	−0.002745	0.9823	0.08539	0.4603	−0.07036	0.4021	−0.05455	0.6611	0.01146	0.9212	0.02573	0.7586	0.142	0.2479	−0.1652	0.151
SBP (mmHg)	−0.05281	0.5296	−0.0619	0.6188	0.02528	0.8272	−0.03399	0.6869	0.02941	0.8146	−0.01608	0.8896	−0.003812	0.9638	0.01348	0.9138	−0.07918	0.4937
DBP (mmHg)	0.003027	0.9713	0.04931	0.6919	0.00927	0.9362	0.1314	0.1177	0.2753	0.0253^*^	0.1125	0.3298	−0.1239	0.139	−0.2005	0.1038	−0.1116	0.334
blood glucose (mmol/L)	0.02941	0.7254	−0.05036	0.6834	0.1562	0.1749	0.06156	0.4636	0.07021	0.5723	0.1076	0.3516	−0.0487	0.5608	−0.05019	0.6844	−0.06732	0.5607
LDL (mmol/L)	0.1202	0.15	0.1837	0.1338	0.021	0.8561	0.1871	0.0248^*^	0.3412	0.0047^*^	−0.02227	0.8475	−0.1189	0.1542	−0.06988	0.5712	−0.1128	0.3288
HDL (mmol/L)	0.08715	0.2973	−0.01778	0.8856	0.1421	0.2175	0.05142	0.5405	0.02043	0.8697	0.118	0.3066	0.1025	0.2198	0.09004	0.4653	0.1413	0.2202
Triglycerides (mmol/L)	−0.09376	0.262	0.02197	0.8589	−0.2695	0.0178^*^	0.0108	0.8978	0.1143	0.357	−0.1761	0.1256	0.03195	0.7029	0.1004	0.4151	−0.002214	0.9848
TG (mmol/L)	0.04346	0.6037	0.1376	0.2632	−0.06205	0.5919	0.1574	0.059	0.3605	0.0027^*^	−0.05137	0.6572	−0.06475	0.4391	0.005444	0.9649	−0.1421	0.2176

*Note*: * means that *p*‐value ≤ 0.05, and the data in bold are statistically significant.

Abbreviations: DBP, Diastolic blood pressure; SBP, systolic blood pressure; TG, total cholesterol.

## 4 Discussion

Our study demonstrates that patients with better baseline health conditions prior to COVID‐19 infection exhibit superior cognitive function postinfection. Additionally, better preinfection health status significantly reduces hospitalization duration, suggesting that overall physical health plays a crucial role in determining post‐COVID‐19 outcomes and cognitive function.

Previous studies have predominantly focused on changes in health status following COVID‐19 hospitalization, including alterations in cognitive function, mental health, and physical condition (Borel et al. 2022; Crivelli et al. 2022). These studies primarily emphasize the impact of COVID‐19 itself rather than preexisting health conditions. Many investigations have also explored factors influencing COVID‐19 severity, such as age, gender, race, and molecular‐level markers like ACE2 (angiotensin‐converting enzyme 2) expression (Palaiodimos et al. 2020; Saatci et al. 2021; Zhang et al. 2023). Furthermore, research has identified hypertension, obesity, and genetic predispositions as risk factors for severe COVID‐19 symptoms (Cruz et al. 2022; de Siqueira et al. 2020; Pranata et al. 2020). However, these factors are largely immutable in the short term.

In contrast, our study incorporates an assessment of pre‐COVID‐19 health conditions, aiming to determine their influence on disease severity and cognitive function postinfection. While previous research has examined postinfection health outcomes, our findings emphasize the impact of modifiable preinfection factors, highlighting the potential for proactive health management to improve postinfection prognosis.

Our results indicate that baseline health conditions can predict both the severity of COVID‐19 and cognitive function following infection. Patients with poorer baseline health experienced more severe disease progression, likely due to compromised immune function and a higher prevalence of underlying conditions. Preexisting conditions, such as hypertension, cardiovascular disease, cerebrovascular disease, and respiratory illnesses, have been widely recognized as contributors to adverse COVID‐19 outcomes (Du et al. 2020; Ren et al. 2022). In our study, we also performed a categorical *t*‐test analysis on cognitive‐related underlying diseases and obtained corresponding significant results, which could also verify previous studies to a certain extent. However, in addition to hypertension, cardiovascular disease, cerebrovascular disease, and respiratory illnesses, the results of all underlying diseases indicate that there are more underlying diseases that can affect the prognosis of COVID‐19 patients, which may lead to a worse prognosis for COVID‐19 patients by affecting their health status. At the same time, these results further support this association by demonstrating a significant correlation between daily health status, underlying diseases, and COVID‐19 severity.

### 4.1 Cognitive Function and MoCA Subdomains

Analysis of MoCA subdomains revealed that orientation and abstraction were not significantly correlated with health scores. Prior studies comparing COVID‐19 patients to healthy controls have reported mixed findings, with some studies showing no significant differences in memory, visuospatial ability, and orientation, while others have highlighted deficits in executive function, language, and attention (Hadad et al. 2022). Our results align with the latter, suggesting that attention and executive dysfunction are key cognitive domains affected by COVID‐19.

Similar patterns of cognitive decline have been observed in other respiratory diseases. For example, patients with obstructive sleep apnea‐hypopnea syndrome have shown MoCA deficits primarily in attention and executive function (Y. Liu et al. 2021). Likewise, aspiration pneumonia has been associated with cognitive decline, with the number of pneumonia‐related hospitalizations correlating with moderate to severe cognitive impairment (Davydow et al. 2013; Girard et al. 2018; Naruishi et al. 2018). These findings support our hypothesis that preexisting health conditions may contribute to cognitive impairment post‐COVID‐19, particularly in attention and executive domains.

Meanwhile, abnormal electroencephalogram (EEG) signals, such as lower EEG delta band at baseline, have also been reported in COVID‐19 patients, which is also associated with cognitive decline (Cecchetti et al. 2022). However, the correlation between EEG and cognitive function was not explored in our study, and further analysis is needed to verify this.

Our results further suggest that underlying diseases significantly impact attention and language functions, which are among the most affected cognitive domains in COVID‐19 patients. This finding underscores the importance of maintaining good overall health to mitigate cognitive decline postinfection.

### 4.2 Potential Mechanisms of Cognitive Decline

Our analysis indicates that deteriorating baseline health status is associated with worse cognitive outcomes post‐COVID‐19, likely due to underlying diseases. Additionally, some biochemical markers measured during hospitalization showed correlations with cognitive status, suggesting that nonneurological organ damage may contribute to cognitive impairment. However, our study does not provide data on whether these cognitive deficits are reversible or indicative of permanent brain damage.

Previous research has proposed several mechanisms linking COVID‐19 to cognitive impairment. *Neuroinflammation*: COVID‐19 may trigger inflammatory responses in the brain, leading to metabolic dysfunction in affected regions (Hosp et al. 2021). *Vascular involvement*: Cognitive decline may be driven by inflammation‐induced vascular damage (Uginet et al. 2021). *Hypoxia*: Oxygen deprivation during infection can contribute to neuronal injury and cognitive impairment (Xiong et al. 2021). *Direct viral invasion*: SARS‐CoV‐2 may directly infect brain tissue and immune cells, further exacerbating cognitive deficits (Bullen et al. 2020).

Our findings reinforce the hypothesis that preexisting health conditions influence cognitive outcomes post‐COVID‐19. Since daily health status reflects an individual's underlying disease burden, routine health assessments could help predict cognitive vulnerability in COVID‐19 patients and provide an early warning for potential cognitive decline.

Biomarker analysis in COVID‐19 patients has shown that most individuals exhibit normal or reduced white blood cell counts, with lymphopenia being a common finding. White blood cell levels tend to be slightly higher in severely ill patients compared to those with milder cases, while platelet counts have shown inconsistent results (K. Liu et al. 2020). Biochemical markers indicative of liver and kidney function damage also increase, with more severe elevations correlating with worse outcomes. The most significant change is observed in LDH (lactate dehydrogenase), followed by AST (aspartate aminotransferase), ALT (alanine aminotransferase), CK (creatine kinase), and creatinine (Guan et al. 2020). Additionally, concentrations of LDH, ALT, AST, creatinine, CK, cardiac troponin I, and N‐terminal pro‐brain natriuretic peptide are significantly higher in deceased patients than in those who recover (T. Chen et al. 2020; Z. Chen et al. 2021; Zhou et al. 2020). SARS‐CoV‐2 may invade the central nervous system (CNS) by binding to endothelial cells and crossing the blood–brain barrier via leukocyte trafficking or sluggish microcirculatory flow. Once within the CNS, the virus can infect ACE2‐expressing cells, including neurons, astrocytes, and oligodendrocytes, then trigger a localized immune response that may contribute to cognitive impairment (Battaglini et al. 2022). Consequently, persistent elevations in key biochemical indicators—such as LDH, CK, AST, ALT, urea, and creatinine—serve as strong predictors of critical illness and poor prognosis. In our study, we found that albumin, white blood cells, and triglycerides were significantly correlated with cognitive function and hospitalization duration. Additionally, albumin was associated with preinfection health status, suggesting that these biomarkers may be valuable in predicting disease severity and patient outcomes.

Numerous studies have sought to identify factors influencing COVID‐19 severity to develop strategies for improving patient prognosis (Bellou et al. 2022; Pillay et al. 2022). Our research contributes to this effort by providing evidence that maintaining good health prior to infection can mitigate both disease severity and cognitive impairment.

The key takeaway from our study is that baseline health status plays a crucial role in postinfection outcomes. Given the ongoing risk of reinfection and the continued global burden of COVID‐19, maintaining overall health should be a priority to reduce severe complications and preserve cognitive function. Future research should further explore specific health indicators that have the greatest impact on COVID‐19 outcomes, allowing for more targeted interventions to improve patient prognosis and minimize long‐term health consequences.

### 4.3 Strengths and Limitations of This Study

This study focuses on pre‐COVID‐19 influencing factors, specifically patients' baseline health conditions, which are modifiable. This provides actionable guidance for individuals to enhance their health and resilience against COVID‐19.

The analysis incorporates not only the severity of COVID‐19 infection but also postinfection cognitive function, offering a comprehensive assessment of the long‐term impact of preexisting health conditions.

The cases included in this study were collected directly from hospitals, ensuring high reliability and data integrity.

The severity of COVID‐19 infection was assessed based on the length of hospital stay, without considering ventilation and oxygen therapy requirements. This may limit the comprehensiveness of the severity assessment. As PSM analysis could not reveal the relationship between variables, a larger and better sample size may be needed for further analysis

## 5 Conclusion

This study highlights the impact of patients' baseline health conditions—modifiable factors that individuals can improve—on post‐COVID‐19 outcomes, including disease severity and cognitive function. Our findings provide valuable insights into mitigating the adverse effects of COVID‐19 by promoting better overall health prior to infection. The results of this study suggest that the health status of COVID‐19 patients can be used to evaluate their prognosis in clinical practice and provide directions for assessing basic health conditions for future research or public health strategies.

## Author Contributions

**Kang Yuan**: methodology, conceptualization, software, writing – review and editing, writing – original draft, formal analysis, validation, visualization, data curation. **Wenbiao Xian**: writing – review and editing, supervision, software, methodology, conceptualization, funding acquisition. **Lishan Lin**: supervision, conceptualization, funding acquisition. **Fengjuan Su**: funding acquisition, investigation, supervision, and conceptualization. **Feiwen Huang**: supervision, funding acquisition, resources. **Wenli Sheng**: supervision, conceptualization, methodology, funding acquisition, writing – review and editing. **Wanlin Wu**: writing – review and editing, conceptualization, investigation, data curation, supervision, resources.

## Peer Review

The peer review history for this article is available at https://publons.com/publon/10.1002/brb3.70793.

## Data Availability

The data that support the findings of this study are available from the corresponding author upon reasonable request.
